# A Pharmacist and Health Coach–Delivered Mobile Health Intervention for Type 2 Diabetes: Protocol for a Randomized Controlled Crossover Study

**DOI:** 10.2196/17170

**Published:** 2021-03-10

**Authors:** Lisa Kay Sharp, Alana Biggers, Rosanne Perez, Julia Henkins, Jessica Tilton, Ben S Gerber

**Affiliations:** 1 Department of Pharmacy Systems, Outcomes & Policy, College of Pharmacy University of Illinois at Chicago Chicago, IL United States; 2 Department of Medicine Section of Academic Internal Medicine & Geriatrics University of Illinois at Chicago Chicago, IL United States; 3 Department of Pharmacy Practice, College of Pharmacy University of Illinois at Chicago Chicago, IL United States

**Keywords:** mHealth, type 2 diabetes mellitus, community health workers, clinical pharmacists

## Abstract

**Background:**

Aggressive management of blood glucose, blood pressure, and cholesterol through medication and lifestyle adherence is necessary to minimize the adverse health outcomes of type 2 diabetes. However, numerous psychosocial and environmental barriers to adherence prevent low-income, urban, and ethnic minority populations from achieving their management goals, resulting in diabetes complications. Health coaches working with clinical pharmacists represent a promising strategy for addressing common diabetes management barriers. Mobile health (mHealth) tools may further enhance their ability to support vulnerable minority populations in diabetes management.

**Objective:**

The aim of this study is to evaluate the impact of an mHealth clinical pharmacist and health coach–delivered intervention on hemoglobin A_1c_ (HbA_1c_, primary outcome), blood pressure, and low-density lipoprotein (secondary outcomes) in African-Americans and Latinos with poorly controlled type 2 diabetes.

**Methods:**

A 2-year, randomized controlled crossover study will evaluate the effectiveness of an mHealth diabetes intervention delivered by a health coach and clinical pharmacist team compared with usual care. All patients will receive 1 year of team intervention, including lifestyle and medication support delivered in the home with videoconferencing and text messages. All patients will also receive 1 year of usual care without team intervention and no home visits. The order of the conditions received will be randomized. Our recruitment goal is 220 urban African-American or Latino adults with uncontrolled type 2 diabetes (HbA_1c_ ≥8%) receiving care from a largely minority-serving, urban academic medical center. The intervention includes the following: health coaches supporting patients through home visits, phone calls, and text messaging and clinical pharmacists supporting patients through videoconferences facilitated by health coaches. Data collection includes physiologic (HbA_1c_, blood pressure, weight, and lipid profile) and survey measures (medication adherence, diabetes-related behaviors, and quality of life). Data collection during the second year of study will determine the maintenance of any physiological improvement among participants receiving the intervention during the first year.

**Results:**

Participant enrollment began in March 2017. We have recruited 221 patients. Intervention delivery and data collection will continue until November 2021. The results are expected to be published by May 2022.

**Conclusions:**

This is among the first trials to incorporate health coaches, clinical pharmacists, and mHealth technologies to increase access to diabetes support among urban African-Americans and Latinos to achieve therapeutic goals.

**International Registered Report Identifier (IRRID):**

DERR1-10.2196/17170

## Introduction

### Background

Diabetes disproportionately affects African-American and Latino adults in the United States compared with Whites [1]. Not only is the prevalence of type 2 diabetes approximately 1.5 times greater, but diabetes-related outcomes are consistently worse. Aggressive management of blood glucose, blood pressure, and cholesterol through medication and lifestyle adherence is necessary to minimize adverse health outcomes [2,3]. However, fewer than 20% of African-Americans and Latinos reach all therapeutic goals [4-11]. Adherence to medication is generally poor [12]: 20%-30% of prescriptions are never filled (due to cost or concerns of side effects), and 50% of medications for chronic disease are not taken as prescribed [13-15]. Additional barriers to physical activity [16] and healthy eating [17] behaviors are common within urban neighborhoods where low-income African-Americans and Latinos reside. Access to healthy foods and safe areas for physical activity are often limited. As a result, adherence to the recommended diet and physical activity is low for a majority of minorities with type 2 diabetes [18,19]. Additional barriers to self-management among low-income, minority populations include low health literacy, depression, lack of social support, poor patient-clinician communication, limited access to health care, and language (particularly Latinos who prefer Spanish for communication with providers) [20-27].

Four large systematic reviews of intervention studies aimed at improving medication adherence have concluded that few existing interventions improved clinical outcomes beyond adherence alone [13,14,28,29]. In addition, the reviews highlighted concerns regarding the abundance of studies with small sample sizes and the underrepresentation of ethnic minorities, especially Latinos. Components of the most effective interventions often included behavioral strategies and incorporated pharmacists [30]. Pharmacist-led interventions enhance adherence to chronic disease medication and health behaviors through patient education, use of adherence aids, and addressing medication-related issues (eg, drug interactions and cost) [14,31-36]. Clinical pharmacists have expertise in medication management and the ability to adjust therapy in collaboration with providers [37-42]. Within the United States, most state boards of pharmacy authorize collaborative drug therapy management protocols between clinical pharmacists and prescribers [42-45].

More recently, 2 pragmatic studies of chronic disease management employed multifaceted interventions delivered by clinical pharmacists using telephone-based brief negotiated interviewing, a variant of motivational interviewing [46,47]. First, Choudhry et al [46] studied patients within a large multispecialty medical group including 488 patients with diabetes and others with hypertension or hyperlipidemia that were poorly controlled. A statistically significant improvement in medication adherence failed to translate into any significant improvement in clinical outcomes for patients with diabetes or the other chronic conditions. In the second study, Lauffenburger et al [47] enrolled members of the largest health insurer in New Jersey with similar results. However, in a secondary analysis of 196 participants with diabetes who completed at least one telephone pharmacy consultation, hemoglobin A_1c_ (HbA_1c_) decreased (difference between intervention and propensity score matched comparison group: mean decrease in HbA_1c_−0.48 with a 95% CI of −0.91, −0.05). Although both studies included ethnic minorities, subgroup analyses to explore the impact of the intervention based on ethnicity were not reported.

Health coaches (HCs) may extend the ability of clinical pharmacists to support medication adherence in low-income minority populations. HCs alone have been shown to contribute to improvements in diabetes self-management and HbA_1c_ levels [48-55]. Trained HCs are trusted by patients, understand sociocultural barriers, and can help increase the relevancy of disease self-management to individuals who manage competing priorities. Our previous research demonstrated that HCs can successfully collaborate with clinical pharmacists in addressing lifestyle and medication adherence [56], with unclear evidence of improved clinical outcomes [15]. In our initial efficacy trial of African-American and Latino patients with uncontrolled type 2 diabetes, we demonstrated a modest reduction in HbA_1c_ with pharmacist support alone (mean decrease in HbA_1c_ −0.45%; with a 95% CI −0.96, 0.05), which was similar to the change observed with HC-augmented pharmacist support (mean decrease in HbA_1c_ -0.42% with a 95% CI −0.93, 0.08). However, many participants did not fully engage with the pharmacists, which required transportation to the clinic for in-person visits. Importantly, 80.4% (152/189) of our study sample surveyed expressed interest in participating in a mobile health (mHealth) approach with pharmacists and HCs.

To address patients’ feedback and enhance our clinical pharmacist and HC support, we designed this study, which incorporates mHealth (text messaging and videoconferencing) combined with in-person HC support. Videoconferencing in diabetes care is practical, potentially cost-effective, and reliable for disease management [46,57,58]. Despite the heterogeneity found in studies conducted in various countries with diverse patient populations, telemedicine interventions produce significantly better glycemic outcomes than usual care [59]. Videoconferencing with clinical pharmacists to promote medication adherence is a growing model in practice but is not well studied, particularly with mobile devices or involving urban, low-income minority patients [60,61]. Our inclusion of HCs to assist pharmacists in the delivery of videoconferencing services is further aimed at supporting adherence efforts and improving outcomes. In addition, HCs use text messaging to provide social support and improve self-efficacy and adherence. Text messaging interventions in diabetes management have shown encouraging results, targeting both medication adherence and lifestyle modification with a reduction in HbA_1c_ [60-64]. Although no specific text messaging components have been linked to improved outcomes, follow-up contact, individualized frequency, and tailored content are likely important components [65,66].

Drawing upon the need to develop new models of care that address the needs of those most adversely impacted by diabetes, our team has developed an innovative clinical pharmacist and HC mHealth model to improve HbA_1c_ (primary outcome). This model targets African-Americans and Latinos (both English- and Spanish-speaking) with uncontrolled diabetes [20,56]. This paper describes our currently implemented mHealth study protocol and includes a crossover randomized controlled trial.

### Objectives

The aim of this study is to evaluate the impact of an mHealth clinical pharmacist and HC intervention in African-Americans and Latinos with poorly controlled type 2 diabetes. We hypothesize that the mHealth intervention will improve HbA_1c_, blood pressure, and low-density lipoprotein (LDL) as well as medication and lifestyle behavior adherence compared with usual care. In addition, the crossover design will test the hypothesis that improvements in outcomes resulting from the intervention in year 1 will be maintained during the maintenance period (usual care) in year 2.

## Methods

### Study Design

**Figure 1:**
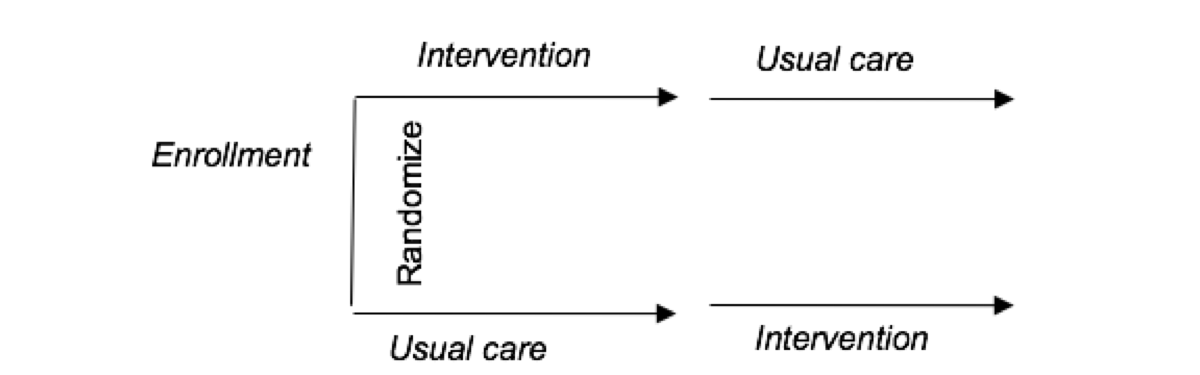
Randomized crossover study design.

As shown in Figure 1, a randomized controlled crossover study will evaluate the effectiveness of an mHealth intervention delivered by a clinical pharmacist and HC versus a usual care group.

The primary outcome is the change in HbA_1c_ level. Secondary outcomes include changes in blood pressure and LDL cholesterol levels. Using a 1:1 ratio, we will randomize 220 patients to either (1) a mHealth intervention delivered by a clinical pharmacist and HC for 1 year, followed by usual care for 1 year, or (2) usual care for 1 year, followed by the mHealth intervention for 1 year. Both groups will crossover at the end of year 1, such that those receiving the mHealth intervention during the first year will receive usual care during the second year (to evaluate potential maintenance of outcome improvement). Similarly, those with usual care in year 1 will crossover to receive the intervention in year 2 (to ensure that all subjects are able to receive the intervention). Of note, we do not consider HbA_1c_ levels collected at the end of year 1 when crossover occurs. The treatment received by patients (intervention or usual care) will change at the end of year 1, independent of HbA_1c_ levels.

Separate randomization schedules generated in REDCap will be used for each of the 4 main clinical sites with stratification for ethnic group (African-American and Latino) and gender to balance the proportion of participants across randomized conditions. All study procedures have been approved by the University of Illinois at Chicago Institutional Review Board (IRB 2016-0380).

### Setting and Recruitment

The study will be conducted at the University of Illinois Hospital and Health Sciences System (UI Health), which includes both inpatient and outpatient facilities serving an urban, largely minority population in Chicago. All outpatient sites share access to the electronic medical record (EMR), Cerner Powerchart.

An initial pool of potential patient participants will be identified through EMR queries and include all patients registered in 4 UI Health internal medicine or family practice clinics. Patients with a documented diagnosis of type 2 diabetes and HbA_1c_≥8 in the last year will be targeted for recruitment. The study team will mail study introductory letters by post to those patients identified from the EMR queries meeting the two initial criteria. The letter explains that if the patient is not interested in being contacted by telephone regarding the study, they should either mail back the enclosed prepaid card or call the telephone number provided to indicate that they are not interested in being contacted further (*opt-out*). Individuals who do not *opt out* are called by a research assistant (RA) to determine study interest and assess eligibility. Eligible and interested patients are scheduled to complete written consent, Health Information Portability and Accountability Act (HIPAA) authorizations, and baseline data collection with a data collector at the Clinical Research Center at UI Health. Additional recruitment is completed by RAs present within the clinics who receive referrals directly from the staff. A study physician reviews patients’ EMR to confirm eligibility (Textbox 1).

**Inclusion and exclusion criteria.**

**Inclusion criteria:**

- Self-identified as Latino or Hispanic or African-American
- Verbal fluency in English or Spanish
- Latest hemoglobin A_1c_ ≥8.0% (within 6 months)
- History of type 2 diabetes (>1 year)
- Aged between 21 and 75 years
- Mobile phone or text messaging plan
- Agrees to home visits by health coach
- Receives primary care at clinical site (>1 year)

**Exclusion criteria:**

- Unable to verbalize comprehension of study or impaired decision making (eg, dementia)
- Lives outside Chicago (≥3 months/year)
- Household member already participating in same study
- Plans to move from the Chicago area within the next year
- Pregnant or trying to get pregnant
- Unable to send or read text message on mobile phone
- History or planned gastric bypass or transplant surgery

### mHealth Intervention

#### Clinical Pharmacist

During the intervention year, patients receive pharmacist videoconferences facilitated by an HC who is in the patient’s home with a tablet (iPad with cellular plan). The initial pharmacist encounter is scheduled after the HC has already conducted 1-2 home visits and lasts 60 minutes. The pharmacist initially reconciles medications via videoconferencing with HC assistance. Subsequent pharmacist encounters vary in frequency based on patient needs and range in length from 30 to 60 minutes. HCs schedule videoconference appointments during dedicated days or times when pharmacists are available. Videoconferences are conducted using VSee software. VSee transmits personal health information securely and is available for use on desktop computers and mobile devices providing real-time person-to-person audio and video communications. However, it is most suitable for health-related communication, as it is Food and Drug Administration registered and HIPAA compliant (using FIPS 140-2 certified 256-bit Advanced Encryption Standard). Finally, it uses *peer-to-peer* sessions so that information is not stored on a server.

Pharmacist services are based on a standardized pharmacist management protocol. After the initial medication reconciliation, follow-up pharmacist activities include reviewing home glucose and/or blood pressure, monitoring log data obtained by the HC during home visits, identifying therapeutic goals for HbA_1c_ and blood pressure collaboratively with patients’ primary care providers (PCPs), formulating an approved plan of care, assessing changes in medications, and documenting the plan in the EMR. In addition, pharmacists provide education related to medication (name and purpose of medications and time, strength, and method of administration); drug interactions and side effects; goals of therapy; basic lifestyle modifications; and use of pillboxes, low-literacy visual medication lists, or other adherence aids. Pharmacists educate and encourage lifestyle changes, consistent with the published guidelines [67-69]. They propose medication changes based on algorithms and protocols derived from national guidelines under physician guidance [70-72]. Pharmacists routinely monitor hypoglycemic events, address prevention, and review treatments. This includes 3 steps: (1) addressing hypoglycemia with every patient contact, (2) applying principles of appropriate therapy (education, empowerment, frequent glucose self-monitoring, flexible medication regimen, individualized goals, and professional guidance), and (3) considering risk factors for hypoglycemia. Overall, there is mixed evidence regarding the benefits of aggressive glycemic control [73-75]. In the proposed study, PCPs and pharmacists adopt the American Diabetes Association approach to individualized care, where the general goal for nonpregnant adults is HbA_1c_ less than 7%. They may decide upon less stringent goals for those with a history of severe hypoglycemia, limited life expectancy, advanced complications, or extensive comorbid conditions [76]. In addition, pharmacists follow the 2018 American College of Cardiology or American Heart Association guidelines for lipid management (eg, calculating 10 year atherosclerotic cardiovascular risk to determine statin intensity) [77].

#### EMR Documentation and PCP Communication

PCPs and pharmacists communicate routinely regarding patient care and are located in the same area within the medical setting. Pharmacists review EMRs, including blood test results, clinical progress notes, problem and medication lists, drug allergies, hospitalization records, and emergency room reports. Nonurgent communication and electronic progress notes from each pharmacist encounter will be sent to the PCP through inbox messaging and note forwarding within the EMR. Pharmacist progress notes include a detailed list of medications, estimated adherence levels, and home glucose or blood pressure monitoring log information.

#### Videoconference Training

Videoconferencing procedures in the intervention follow the American Telemedicine Association practice guidelines [68]: (1) pharmacist and patient/HC identity verification, (2) informed consent, (3) appropriate physical environment (privacy, lighting, and noise), (4) education and training (pharmacist and HC), (5) alternate communication (eg, telephone contact in case of disruption of service), and (6) documentation in UI Health EMR (by pharmacist and HC). All study pharmacists and HCs receive standardized training on the use of videoconferencing, which includes scheduling, preparing the environment (home or pharmacist office), patient education on telehealth, and documentation. All encounters have contingency plans in place for technology problems (eg, inadequate signal for video streaming through iPads), including the use of mobile phones for all HCs.

#### HC

HCs will introduce themselves to the patients in person at the data collection visit that aligns with the beginning of the patients’ intervention year (ie, baseline visit or crossover visit at the beginning of year 2 depending on randomization). When possible, the first home visit will be scheduled at the initial introductory meeting. If this is not possible, the HC will follow up by telephone within 1 week to schedule the first home visit. All HCs are either African-American or bilingual or bicultural Latino and work with patients who are concordant for race or ethnicity and language. The HCs have an undergraduate college degree in or related to community health with experience conducting home visits. Specific training for the research study includes 80 hours of standardized HC training or retraining. As outlined in Textbox 2, training begins with an overview of the research protocol and discussion of the HC goals. The fundamental components of providing health support to marginalized populations are addressed with the required reading materials, didactic presentations, and discussions [78].

HC training highlights the unique qualification of HCs to provide culturally sensitive support, tailor visits based on the personal preferences of participants, and assist patients in navigating the health care system [79,80]. Diabetes education follows the Diabetes Education Empowerment Program, which targets literacy and cultural awareness [81], and the Training Curriculum for Health Coaches [52,82]. Training includes shadowing a clinical pharmacist to understand medication use, adherence, glucose and blood pressure monitoring, insulin injection, and medication reconciliation. The study investigators provide training in videoconferencing (eg, scheduling and practice encounters), text messaging (eg, practice with the custom text messaging platform and standardized safety procedures), and conducting home visits in a culturally sensitive manner. Ongoing training is provided periodically on topics that reinforce and expand the initial training (eg, motivational interviewing and insulin management). HCs receive routine orientation to clinical operations and staff at their primary care locations. Standardized safety procedures related to text messaging and home visits are also addressed. To evaluate clinical skills, the HCs demonstrate reliable measurement of blood sugar, blood pressure, and administration of insulin (though HCs do not administer insulin to patients).

The HC component involves monthly home visits with ongoing telephone support, including the facilitation of all pharmacist videoconferences. The HCs work with the pharmacist to evaluate adherence (eg, check label instructions and fill and expiration dates of pill bottles), assist in medication reconciliation, review home glucose and/or blood pressure monitoring data, and provide reinforcement of proper medication use. Finally, HCs debrief patients after pharmacist encounters to reinforce and clarify recommendations and plans. HCs and pharmacists communicate with each other between videoconference encounters by phone or secure email, as needed, to coordinate efforts.

**Summary of health coach training.**

Introduction to research study

- Project history and overview
- Study protocol
- Institutional Review Board and human subjects research training

Role of health coaches (HCs)

- Core competencies and roles of HCs
- Role of HCs in addressing health disparities
- Understanding trauma and supporting survivors

Navigating the health care system

- Primary care clinic workflow
- Navigating electronic medical record and encounter documentation
- Patient advocacy and empowerment
- “Closing the loop,” connecting patients to care

Diabetes education

- Understanding diabetes and risk factors
- Disease pathophysiology, complications

Disease management

- Blood glucose monitoring
- Medication therapies for diabetes
- Medication reconciliation, adherence
- Treating hypoglycemic events
- Nutrition and physical activity education

Health coaching strategies

- Patient-centered collaboration
- Motivational interviewing
- Specific, measurable, achievable, relevant, and time-specific goals
- Stress reduction, coping with depression

Home visits and safety

- Ethical considerations, boundary setting
- Cultural humanity
- Safety guidelines and self-defense

Mobile health technology

- Videoconferencing software (VSee)
- MyTapp text messaging system
- Best practices for telehealth encounters

Home visits include the HC and patient. Family members are permitted by patient requests. Through open discussion and reflective listening, HCs encourage patients to explore their emotions and share their concerns [83]. Overall, HCs provide diabetes self-management education and support (DSME/S), consistent with the recommendations of the American Diabetes Association’s position statement [83]. Specifically, HCs provide DSME/S that includes the following over the course of the 1-year intervention: engagement, information sharing, psychosocial and behavioral support, integration with other therapies, and coordination of care. Initial HC encounters focus on relationship building and gaining an understanding of the patient’s individualized diabetes education needs. HCs provide diabetes education and consider realistic and achievable food choices, portion sizes, and cooking preparation; discuss relationships between medications, meals, and glucose levels; and help patients integrate movement into their daily lifestyles. Diabetes education is facilitated by our culturally appropriate multimedia education iBook, *Living Well with Diabetes/Viviendo Bien con Diabetes* [84]. This iBook (available in English and Spanish) provides patients with video testimonials and various interactive educational experiences. HCs present specific chapters on an iPad to reinforce specific diabetes self-management concepts [84]. To promote behavioral change, HCs use motivational interviewing techniques to assist patients in setting specific health behavior change goals. Goals set jointly by HCs and patients follow the specific, measurable, achievable, relevant, and time-specific (SMART) framework [85]. Providing social support while helping patients engage with their existing support systems offers the potential of long-term behavior change beyond the duration of the research period. Educating patients on how to effectively navigate the health care system and use existing resources promotes continued self-efficacy. In summary, HC interactions are culturally tailored to individual needs, preferences, and resources to provide DSME/S [86].

By the end of the second month, HCs will facilitate a videoconference encounter with a pharmacist and patient to conduct a complete medication reconciliation. HCs work with patients to identify adherence barriers and assist in problem solving or referrals for resources aimed at overcoming recognized challenges in medication use. Finally, HCs document summaries of each encounter (with or without videoconferencing) in the EMR and forward notes electronically to PCPs and pharmacists.

#### Text Messaging

Between home visits, HCs will communicate with patients through telephone calls and text messaging. All text messages will be tailored by HCs and sent through a custom software app (*mytapp*). We developed *mytapp* for community-based health behavior research. *mytapp* sends messages immediately or at a scheduled date or time, recurrent messages (daily, weekly, or weekdays), group messages, or multiple question surveys. HCs receive training on *mytapp* and schedule messages for their patients regarding appointments and medications. HCs send messages to maintain motivation, elicit feedback on progress, and screen for barriers that may reduce the chance of success (Table 1). For example, a morning text message may ask a patient how they did on their goal the previous day.

No more than 7 messages are delivered weekly, except for optional medication reminders. For safety, patients are reminded that text messages are sent in an automated fashion by a computer. Furthermore, they are reminded that urgent health questions should be directed to their PCP and not sent in a text message. HCs monitor patient responses through their study phones.

Texts are monitored by the HCs with additional oversight by the project coordinator.

**Table 1:** Example text message templates.

Message type	Example
Medication reminder	“Hi, Ms. Brown, just checking to see if you took your morning pills.”
Refill reminder	“Make sure you don't run out. Check to see when a pill refill is due.”
Appointment reminder	“Just a reminder that you have a doc’s appointment today.”
Goal monitoring	“Remember your goal. Did you take your meds last night?”
Glucose monitoring	“Hello, Mr. Marquez, have you had time to check your sugar today?”
Self-efficacy	“Taking your meds is within your control. You can do it.”
Motivation	“You have come a long way! Keep up the good work!”

#### Usual Care

All participants spend 1 year in the usual care condition, either year 1 or 2, depending on the randomization. During receipt of usual care, they receive health care from their usual providers without the support of a pharmacist or HC. There are no home visits or pharmacist telehealth videoconferencing encounters with usual care. In addition, participants receive a 1-page list of clinic resources with names and direct telephone numbers (eg, social worker or clinical pharmacist) along with a low-literacy, paper-based diabetes education pamphlet [87]. Usual care reflects the type of health care that patients receive outside of any participation in research.

#### Intervention Fidelity

To continuously evaluate the fidelity of both conditions, we review logs maintained by the HCs weekly, including visit dates, length of visits, visit content, and disposition (eg, visit completed, and patient unreachable). Weekly group meetings with HCs and the research team provide an opportunity to monitor intervention delivery. Monthly lunch meetings with clinical pharmacists, HCs, and investigators ensure active collaboration between HCs and pharmacists. All technology-related difficulties are reported to the coordinator immediately and discussed in weekly meetings with the investigators. One investigator, a health psychologist, maintains weekly contact with HCs to provide emotional support and incorporate structured training opportunities. Text messages sent and received via *mytapp* are reviewed monthly.

#### Data Collection

Trained, blinded RAs collect physiological and self-report data through interview administration within the clinical research center at UI Health at 5 time points: baseline and every 6 months for 2 years. RAs are matched to patient language preferences in English or Spanish. Laminated cards with Likert-type scale responses are provided as a visual reference to the patients. Interview data are entered directly into laptop computers using Research Electronic Data Capture (Vanderbilt University) electronic data capture web application [88]. The baseline survey requires an average duration of 60 minutes with subsequent follow-up visits lasting 30 minutes. Subjects receive US $30 as compensation for travel and time at each of the 5 data collection points (plus US $50 if at least one videoconference is completed in the prior 6 months during the intervention period). Public transit cards and parking passes are provided when needed. Participants are informed of their randomization assignment at the end of the baseline data collection.

Sociodemographics include age, gender, self-reported race and ethnicity, country of origin, income, highest level of education, employment status, global health status [89], and insurance. Diabetes and medical history include self-reported time since diabetes diagnosis, prior receipt of diabetes education, current therapy, known diabetes complications, and comorbid conditions. Health literacy is assessed using 3 screening questions with high discriminatory power among English- and Spanish-speaking populations [67,90,91]. Mobile phone use and comfort sending text messages is also assessed using 5 items [68].

Intermediate variables are also collected at each of the 5 time points. Perceived severity of diabetes and perceived susceptibility are assessed using 2 items adapted from the study by Bradley et al [92]. The perceived benefits of therapy are measured by a 5-item survey related to the benefits of therapy [71]. Diabetes distress is measured using the brief Diabetes Distress Scale [73]. Depression is measured using the Patient Health Questionnaire [75,76]. Social support is measured using an assessment of the amount of total support received and satisfaction with support from family, friends, and the health care team [93]. Self-efficacy is measured using the Stanford 8-item self-efficacy for diabetes survey [94]. Contextual data include an environmental survey that addresses loneliness, social cohesion, and neighborhood safety as well as the identification of stressful life events [95-97].

HCs record the dates of patient contacts in REDCap (eg, phone calls and home encounters). Clinical progress notes are completed in the EMR after every participant contact (by phone if longer than 15 minutes and in-person). Information includes mode, time, location (home vs clinic), content of contact, results of glucose or blood pressure self-monitoring, goals, life events, pharmacist interactions, and interventions. Intensification of therapy will be defined as the number of dosage increases of antihypertensive, hypoglycemic agent, or insulin or the addition of a new agent since the baseline visit [98-100]. Chart review will define the number of PCP visits and pharmacist videoconferences as well as the number of pharmacist- or physician-initiated medication changes.

*Physiologic outcomes* will be collected at 5 time points (0, 6, 12, 18, and 24 months). The research staff will perform phlebotomy, blood pressure, weight, and height recordings. HbA_1c_ and fasting lipid profile (total cholesterol, high-density lipoprotein, LDL, and triglycerides) are obtained via phlebotomy. A calibrated digital scale measures weight. A height stadiometer measures height, with subjects removing their shoes (for BMI assessment). Blood pressure is assessed in subjects sitting down for at least 5 minutes, following a standard procedure. Health-related quality of life is measured using the EuroQol Group 5D and Diabetes Distress Scale [73].

The revised Summary of Diabetes Self-Care Activities Measure captures basic diet, exercise, blood sugar testing, foot care, and smoking with 11 core items [101]. Additional questions address skipping medications and insulin injections to evaluate adherence, as well as taking aspirin regularly. Alcohol misuse is assessed using the Alcohol Use Disorder Identification Test—Concise [102].

#### Sample Size Justification

The sample size calculation is powered to detect the primary outcome, which is the change in HbA_1c_. Reviews of published studies suggest that successful education programs lower A_1c_ levels by 0.4% to 1.7% [3,103]. On the basis of our previous experience with the patient population, we estimated a mean baseline HbA_1c_ level of 10% with a SD of 1.8 and an effect size of 0.56 for aim 1. The cross-time correlation was estimated to be 0.30, with a compound symmetry structure. We adjusted for clustering and assumed an intraclass correlation coefficient of 0.01, with 5 clusters. This yielded a design effect of 1.34. Two-sided alpha of .05 and 80% power were assumed. Allowing for a 20% dropout rate, 220 patients were required [104].

#### Data Management and Analysis

To address missing data, we will examine the data for patterns of missingness and potential bias in missingness. If data are not missing at random, we will apply one of several available imputation methods in a sensitivity analysis based on the nature and extent of missing data [105]. We will determine effectiveness using intention-to-treat principles with actual imputation of missing data [106-109]. This will allow us to appreciate the potential biases inherent in a *real-world* setting due to dropout and poor adherence to study procedures.

Patients who share a single PCP might exhibit similarities that are not shared with patients cared for by other PCPs. To address this, we will include random effects in the model for clinic site, PCP, and HC (though the small numbers of HCs may call for a fixed effect approach or *insufficient replication*) [110]. To examine a stricter examination of effect, we will also do a *per-protocol* analysis of complete cases. Completed cases will not be dependent on intermediate time point data collections (6 and 18 months). Potential selection bias will be corrected by including model covariates that are differentially related to study participation.

Univariate comparisons between the 2 study groups for outcomes and covariates at baseline will be conducted using chi-square tests for categorical variables, Kruskal-Wallis tests for ordinal variables, and *t* tests for continuous variables. All tests will be two-sided. Nonnormal continuous data will be transformed before the analysis. To provide a comprehensive analysis of our primary hypothesis, we will extend the usual analysis of crossover designs [111] by including a longitudinal trend component in the first year. Thus, we can examine the time course (0, 6, and 12 months) as linear or quadratic over the first 3 measurements. This will allow us to investigate whether changes are made early and at what rate they continue throughout the rest of the period. This analysis permits a comparison of the trends between the 2 conditions. In addition, we will regard subjects as a random effect and will use Gaussian mixed model estimation. We can then substitute treatment by period interactions for the carry-over effects, and the model can be reduced in a recommended sequence (first omit carry-over, then omit treatment, and finally omit period) [111]. We will also explore patterned covariance structures such as compound symmetry and autoregression along with incorporating time-constant and time-varying covariates, such as health literacy or diabetes distress.

The primary analysis of all physiological outcomes jointly in the repeated measures design will be conducted using a general linear model framework. Repeated measures multivariate analysis of variance will be used to explore the simultaneous impact of the treatment on multiple correlated dependent variables, including the use of Roy-Bargmann stepdown *F* tests and discriminant function analysis as post hoc tests to identify subsets of outcome measures affected [112]. Multivariate analysis of variance (MANOVA) secondary analyses will explore the impact of inhomogeneous baseline variables on the results. Group by time-trend interaction contrasts will be used to explore different group trajectories of change. The potential consequences of medication intensification (eg, initiation of insulin) will be explored by evaluating changes in BMI and quality of life.

Exploratory subgroup analyses will follow a heterogeneity in treatment effects framework [113,114]. We will determine which subjects in the intervention group had the greatest improvement in outcomes, based on multiple prespecified patient characteristics, including race (African-American or Latino), depression, comorbidities, baseline behaviors, health literacy, continuity of care, and social support. These analyses will also consider additional comparisons between subjects above and below the median levels of videoconferencing and text messaging activity. Statistical tests will be implemented as interactions within the full data set.

If the intervention results in improved HbA_1c_ more than that in the usual care, we will examine whether changes in self-efficacy, health beliefs, or social support serve as mediators for improved outcomes. In addition, we will explore diabetes-related behaviors as well as medication treatment intensification as mediators using MPlus [115-118]. To further identify the relative contributions of videoconferencing and/or text messaging, we will conduct meditational modeling with videoconferencing time, number of text messages, and HC contact time as mediating variables. This may describe any dose-response relationship between mHealth utilization and outcomes. We will compute bias-corrected bootstrap standard errors using MPlus. This offers accurate confidence intervals for mediation coefficients [119]. Due to anticipated relationships between HC activity level and mHealth delivery, we plan to inspect correlations between mediators and incorporate any significant findings in model development. This analysis will demonstrate whether the intervention effects are sensitive to mediators. Finally, given the sample size, observed (rather than latent) variables will be used in the mediation models. To enhance the power of mediation modeling, we adopt an α=.10 type I error criterion to improve the chances of finding promising mediators for future studies.

We expect a limited amount of decline in physiologic outcomes with the transition back to usual care. We will test the hypothesis that improvements in HbA_1c_ (primary outcome), blood pressure, and LDL cholesterol (secondary outcomes) will be maintained during the maintenance period. The analyses will be conducted in a manner similar to our primary hypothesis and will evaluate changes in the initial intervention group 1 year after intervention completion.

## Results

**Figure 2:**
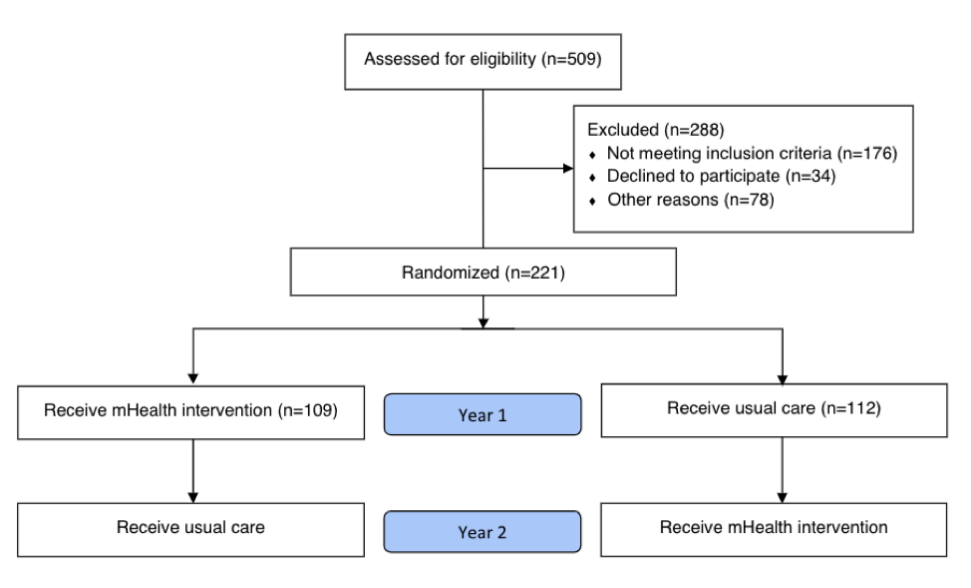
Consolidated Standards of Reporting Trials flow diagram. mHealth: mobile health.

The study was initiated in July 2016, and enrollment began in March 2017. Figure 2 shows the Consolidated Standards of Reporting Trials diagram for recruitment.

As shown in Table 2, 221 patients have been enrolled, exceeding our goal of 220 patients. A total of 112 patients have been randomized to usual care for their first year, and 109 patients have been randomized to the mHealth intervention.

The retention rate is currently 90% for subjects completing the first year of the study. We anticipate that data collection will be completed in November 2021. Following data analyses, the manuscript will be developed with primary results by May 2022 for peer-reviewed publication.

**Table 2:** Demographic characteristics of study population.

Characteristic		Usual care (n=112)	Intervention (n=109)
Age (years), mean (SD)		54.5 (9.6)	56.0 (9.3)
Diabetes duration (years), mean (SD)		12.3 (7.9)	13.1 (7.7)
**Ethnicity, n (%)**			
	Latino or Hispanic	36 (32.1)	37 (33.9)
	African-American	76 (67.9)	72 (66.1)
**Gender, n (%)**			
	Male	35 (31.2)	32 (29.4)
	Female	77 (68.7)	77 (70.6)
**Language preference, n (%)**			
	English	97 (86.6)	87 (79.8)
	Spanish	15 (13.4)	22 (20.2)
**Income (US** $**), n (%)**			
	Less than 10,000	38 (33.9)	36 (33.0)
	10,000-19,999	19 (17.0)	26 (23.8)
	20,000-29,999	12 (10.7)	16 (14.7)
	30,000-39,999	7 (6.2)	10 (9.2)
	40,000-49,999	12 (10.7)	0 (0)
	50,000-59,999	6 (5.4)	8 (7.3)
	60,000-69,999	2 (1.8)	3 (2.8)
	70,000 or more	14 (12.5)	8 (7.3)
	Refused to answer	2 (1.8)	2 (1.8)
**Education, n (%)**			
	Less than high school	26 (23.6)	29 (26.6)
	High school diploma or equivalent	22 (19.6)	33 (30.3)
	Some college, 2-year certificate, or associates degree	41 (36.6)	26 (23.8)
	College graduate (4 year)	11 (9.8)	13 (11.9)
	Some graduate school	3 (2.7)	3 (2.8)
	Graduate degree	8 (7.1)	5 (4.6)
	Other	1 (0.9)	0 (0)
**Health status, n (%)**			
	Excellent	2 (1.8)	0 (0)
	Very good	4 (3.6)	5 (4.6)
	Good	42 (37.5)	38 (34.9)
	Fair	47 (42.0)	58 (53.2)
	Poor	17 (15.2)	8 (7.3)
**Insurance, n (%)**			
	None	5 (4.5)	8 (7.3)
	Public	69 (61.6)	70 (64.2)
	Private	36 (32.1)	30 (27.5)
	Other	2 (1.8)	1 (0.9)

## Discussion

This study assesses the effectiveness of a novel mHealth approach for improving diabetes self-management in an underserved, minority population with type 2 diabetes. The study will provide further evidence of the use of mHealth in both clinical and community environments, with tools to support clinical pharmacists and HCs in their patient-oriented activities. If effective, this intervention can be considered for implementation in low-resource settings where pharmacist services are not readily available, and HCs can extend the reach of providers targeting patients with limited access to care.

### Strengths and Limitations

The study is innovative in several ways. The proposed study will be the first randomized controlled trial to evaluate an mHealth intervention to improve diabetes management in low-income African-Americans and Latinos with type 2 diabetes delivered through HCs. This study builds upon prior work and is responsive to patient feedback in offering remotely delivered videoconferencing with clinical pharmacists who collaborate with HCs to improve diabetes outcomes [56]. Pharmacist videoconferencing overcomes the transportation barriers commonly experienced by individuals with limited income. We currently use scalable, inexpensive mHealth tools (VSee and *mytapp*) to promote adoption in low-resource clinical organizations, including Federally Qualified Health Centers.

A number of limitations and challenges have been identified. First, there is the potential for contamination across groups, as randomization was not clustered. Intervention and usual care patients may share the same provider and clinical site. In addition, we recruited patients from a single urban health care system, which may limit generalizability. Additional studies in practice networks would require the availability of clinical pharmacists and HCs. Finally, our intervention integrates multiple components: clinical pharmacists, HCs, videoconferencing, and text messaging, so we are unable to determine the impact of each component individually.

In conclusion, despite the widespread use of mobile devices, little is known about the effectiveness of the technology in improving health care delivery or outcomes. Although systematic reviews show the preliminary value of text messaging, patient education apps, and videoconferencing in chronic disease management, most studies have been small in size, underpowered, and low in quality and include motivated, nonminority subjects. This study will provide the evidence needed on the impact of mHealth diabetes adherence support delivered to low-income minority patients with uncontrolled type 2 diabetes.

## Appendix

Multimedia Appendix 1 Peer-review report by National Institute of Diabetes and Digestive and Kidney Diseases (NIDDK).

## Abbreviations

DSME/S: diabetes self-management education and support
EMR: electronic medical record
HbA1c: hemoglobin A1c
HC: health coach
HIPAA: Health Information Portability and Accountability Act
LDL: low-density lipoprotein
MANOVA: multivariate analysis of variance
mHealth: mobile health
PCP: primary care provider
RA: research assistant
UI Health: University of Illinois Hospital and Health Sciences System
