# Supplementary material for: A Pharmacist and Health Coach–Delivered Mobile Health Intervention for Type 2 Diabetes: Protocol for a Randomized Controlled Crossover Study
Source: JMIR Res Protoc. 2021 Mar 10;10(3):e17170. doi: 10.2196/17170 (PMC7991981; doi:10.2196/17170)
Supplement: Multimedia Appendix 1 [file resprot_v10i3e17170_app1.pdf]

SUMMARY STATEMENT
(Privileged Communication)

PROGRAM CONTACT:
Christine Hunter
301-594-4728
hunterchristine@niddk.nih.gov

Release Date: **02/23/2016 09:40 AM**
Revised Date:

Application Number: **1 R01 DK108141-01A1**

Principal Investigators (Listed Alphabetically):

GERBER, BEN STEVEN (Contact)
SHARP, LISA K

Applicant Organization: UNIVERSITY OF ILLINOIS AT CHICAGO

Review Group: **HDEP**
Health Disparities and Equity Promotion Study Section

Meeting Date: **02/04/2016**
Council: **MAY 2016**
Requested Start: **07/01/2016**

RFA/PA: **PA14-334**
PCC: **DKH BEHV**
Dual IC(s): **NR**

Project Title: **mHealth for Diabetes Adherence Support**

SRG Action: **Impact Score:20 Percentile:8**
Next Steps: **Visit http://grants.nih.gov/grants/next_steps.htm**
Human Subjects: 30-Human subjects involved - Certified, no SRG concerns
Animal Subjects: 10-No live vertebrate animals involved for competing appl.
Gender: 1A-Both genders, scientifically acceptable
Minority: 2A-Only minorities, scientifically acceptable
Children: 3A-No children included, scientifically acceptable
Clinical Research - not NIH-defined Phase III Trial

Project Year	Direct Costs Requested	Estimated Total Cost
1	483,948	776,325
2	487,577	782,146
3	492,106	789,411
4	490,389	786,657
5	502,071	805,397
TOTAL	2,456,091	3,939,935

ADMINISTRATIVE BUDGET NOTE: The budget shown is the requested budget and has not been adjusted to reflect any recommendations made by reviewers. If an award is planned, the costs will be calculated by Institute grants management staff based on the recommendations outlined below in the COMMITTEE BUDGET RECOMMENDATIONS section.

1R01DK108141-01A1 GERBER, BEN

RESUME AND SUMMARY OF DISCUSSION: This application proposes a randomized, controlled trial to implement and evaluate a mHealth diabetes adherence support intervention delivered by clinical pharmacists and health coaches to improve medication adherence, healthy eating, and physical activity behaviors among African-American and Latino adults with uncontrolled type 2 diabetes. The proposed study addresses a significant public health issue among African-Americans and Latinos, many of whom are at high risk for complications given a lack of adherence to treatment recommendations. The project retains the strong features of the previous submission, including the outstanding investigative team, an appropriate cross over design and supportive preliminary data. During discussion, reviewers noted that the resubmission is also strengthened by changes that are responsive to most of the concerns identified in the previous review, including a more compelling rationale for the use of videoconferencing. The panel noted some remaining concerns; however, these were considered minor and detract only minimally from this otherwise compelling project. Overall, reviewers concurred that the proposed research is potentially very important and likely to inform strategies to reduce disparities in diabetes care and have high impact on the field of diabetes management support services.

DESCRIPTION (provided by applicant): Many African-Americans and Latinos with diabetes do not achieve recommended diabetes goals placing them at high risk for complications. Team-based models of care can help in reaching goals of therapy. Additionally, mobile health (mHealth) technologies can further improve outcomes among those more difficult to reach. This study will evaluate the impact of a team-based, mHealth intervention designed to improve medication adherence, healthy eating, and physical activity behaviors. We will compare this mHealth approach with usual care. Clinical pharmacists and health coaches (HC) will deliver our proposed team-based intervention. mHealth delivery includes mobile phone text messaging, secure videoconferencing, and HC home visits. Pharmacists will focus on medication reconciliation and adherence. Health coaches will help identify psychosocial and environmental challenges to adherence in a culturally-sensitive manner. Together, they can assist in goal-setting, problem-solving, negotiation of competing priorities, and provide social support leveraging mHealth technologies. Preliminary data from our research group supports the role of health coaches partnering with clinic- based pharmacists in improving diabetes outcomes in minorities. In the proposed mHealth intervention, patient- pharmacist videoconferencing will eliminate the need for in-person visits with a pharmacist, which is impractical for many low-income patients. In addition, our pilot work suggests that text messaging is a preferable means of communication and may facilitate more frequent contact with patients. We propose a randomized, controlled trial to evaluate the effectiveness of a mHealth diabetes adherence support intervention delivered by clinical pharmacists and health coaches. We will randomize 220 patients through UI Health to either: (1) mHealth diabetes adherence support through clinical pharmacists and health coaches; or (2) usual care. After one year, patients completing the mHealth intervention will be monitored for an additional year while the usual care group receives the mHealth approach. Outcomes include medication adherence, hemoglobin A1c, blood pressure, and LDL-cholesterol levels. The specific aims include: (1) evaluate the effectiveness of an mHealth diabetes adherence support intervention delivered by clinical pharmacists and health coaches to African-American and Latino adults with uncontrolled type 2 diabetes; (2) evaluate the maintenance of improved diabetes behaviors as well as clinical outcomes one year after completing the intervention; (3) evaluate the cost and cost-effectiveness of mHealth diabetes adherence support compared to usual care; and (4) evaluate the reach, adoption, and implementation of mHealth diabetes adherence support based on the RE-AIM framework.

PUBLIC HEALTH RELEVANCE: This research evaluates a mHealth diabetes adherence support intervention delivered by health coaches and clinical pharmacists, designed to improve adherence to medications and a healthy lifestyle. Outcomes measured include change in hemoglobin A1c, blood pressure, and cholesterol levels. This study will determine the benefit and cost of including mobile

phone text messaging, videoconferencing, health coaches, and clinical pharmacists in diabetes management support services.

CRITIQUE 1

Significance: 1
Investigator(s): 1
Innovation: 4
Approach: 4
Environment: 1

Overall Impact: These are very skilled researchers addressing a highly significant problems in both African American and Latino populations. The investigators have been responsive to a previous review. The study design, power and statistical analyses are sophisticated and appropriate, and the process and program evaluation are salient features. Concerns about secondary outcome measures and potential contamination reduce enthusiasm for the proposal, as does the modest amount of innovation. Nevertheless, the combined pharmacist/health coach/mHealth intervention holds promise of success.

1. Significance:
Strengths
- Diabetes is a problem among African-Americans and Latinos, many of whom are at high risk for complications given a lack of adherence to treatment recommendations.
- Clinical pharmacists are underutilized in terms of their potential to promote behavior change for risk reduction in community populations.
- More proven in this regard are health coaches who can be very effective but need an expert's guidance, as is offered through this mHealth intervention.

Weaknesses
- None noted

2. Investigator(s):
Strengths
- This study is being led by Drs. Ben Gerber and Lisa Sharp. Dr. Gerber has an MD as well as an MPH in public health informatics. Dr. Sharp has a PhD in clinical psychology with additional postdoctoral training in health psychology. Dr. Sharp also has a bachelor's degree in nursing and worked for a decade in this area therefore, adding to her qualifications. The two have collaborated on a closely related R01 funded by NIDDK that is concluding this spring. These two and their collaborators comprise a highly qualified team well positioned to carry out this study effectively.
- The PIs and team have relevant research experience working with all technology components of the intervention, as well as conducting research that involves the inclusion of pharmacists and health coaches.

Weaknesses
- None noted.

3. Innovation:
Strengths
- The partnership between clinical pharmacists and health coaches with the addition of mHealth is innovative and unique.

Weaknesses
- The assertion that health coaches using video conferencing is novel can be challenged. For example, Ramirez and her colleagues were using closed circuit TV and *promotoras* 30 years

ago in the Eagle Pass and Rio Grande areas of Texas 3. Of course, the technology was more basic but the theme was the same.

- REAIM and long term follow up are empirical strengths but are not innovations. Generally, this section is overstated.

4. Approach:
Strengths

- The randomized design and especially block randomization to ensure race, gender and site balance is commendable.
- The recruitment and retention plans are detailed, clearly based on past success.
- The sample size estimation and statistical analyses sections are clear and appropriate for the study aims and will include intention-to-treat models.
- Process and program evaluation ideas (e.g. the cost-effectiveness analysis) are also very good.
- Finally, the outcome evaluation emphasizes medication adherence, hemoglobin A-1 C, blood pressure and LDL-cholesterol, very appropriate for this study's aims.

Weaknesses

- The conceptual framework comprises a wide range of theories (e.g. SCT and HBM) which individually would be all inclusive and very non-specific. Motivational Interviewing will be used, with no attempt to weave it into the other models. The interventions seem to be retrofitted onto this overly general framework, yielding a mixture that is really just a kitchen sink approach. Hypotheses are derived from the literature and the authors' own research but not from these theories.
- Detail is needed on the exclusion criterion of 'comprehension' and 'impaired decision making'.
- Will the pharmacist be sure not to 'contaminate' control subjects with intervention-based information?
- 24 hour diet recalls and the IPAQ can be challenged in terms of their reliability and validity, especially with participants who may be at lower levels of literacy and education. Internal consistencies as low as .47 would not generally be adjudged "acceptable" (p. 90).

5. Environment:
Strengths

- The campus and health system environments are excellent.

Weaknesses

- None noted

Protections for Human Subjects:

Acceptable Risks and/or Adequate Protections

- No concerns were identified.

Data and Safety Monitoring Plan (Applicable for Clinical Trials Only):

Acceptable

- No concerns were identified.

Inclusion of Women, Minorities and Children:

- Sex/Gender: Distribution justified scientifically
- Race/Ethnicity: Distribution justified scientifically
- Inclusion/Exclusion of Children under 21: Excluding ages < 21 justified scientifically
- No concerns identified.

Vertebrate Animals:
Not Applicable (No Vertebrate Animals)

Biohazards:
Not Applicable (No Biohazards)

Resubmission:
- The resubmission is largely responsive though concerns remain about the approach and innovation.

Budget and Period of Support:
Recommend as Requested

- No concerns were identified.

CRITIQUE 2

Significance: 1
Investigator(s): 2
Innovation: 3
Approach: 4
Environment: 1

Overall Impact: This resubmission addresses the effectiveness of a mHealth diabetes care adherence support program, delivered by pharmacists and health coaches in African American and Latinos with uncontrolled diabetes. The project focuses on a compelling problem in this high risk and underserved population. The concerns raised in the previous review have been addressed. The project is well described, the investigative team is strong and previous and ongoing studies on the topic further support its merit. Inclusion of the health economist is a particular strength. Concerns include lack of information about how contamination will be prevented, and lack of detail about how motivational interviewing will be integrated. Information regarding the training and expertise (biosketches) of two of consultants was not apparent.

1. Significance:
Strengths
- Diabetes is a significant burden among African-American and Latino populations; interventions to improve self-management adherence are desperately needed.
- Numerous barriers to successful self-management can be overcome potentially with mobile health technologies that promote easier access, communication, and patient education, including text messaging and video conferencing (mHealth).
- Integrating services-clinical pharmacists and health coaches to support self-management.

Weaknesses
- None noted

2. Investigator(s):
Strengths
- A strong and well qualified team has been assembled to carry out the project.
- A health economist has been added to the team as recommended in the prior review.

Weaknesses

- Dr. Fisher (consultant) will provide expertise in sustainable peer support models of diabetes management and Dr. Fairbanks (consultant) will provide training in motivational interviewing related to adherence. However, biosketches were not available nor are they listed in key personnel.

3. Innovation:
Strengths
- Pharmacists and health coaches, videoconferencing and text messaging technology.
- Cost effectiveness analyses.
- Scientific expertise, the issue of sustainability and the RE-AIM framework.

Weaknesses
- The field of mobile health technology and its use in under-served and high risk populations has a history of at least 25 years or more.

4. Approach:
Strengths
- Addresses issues raised in prior review regarding potential overlap with a current study that ends in April, 2016 (i.e. pharmacists and health coaches with no mobile health technology).
- RCT design; further detail about behavioral outcome measures.
- Availability of a low cost two-way text messaging application developed from experience in other studies (i.e. the Moving Forward weight loss intervention).
- Strong pilot work with the target populations.
- Conceptual framework guiding the study that integrates health belief, social cognitive theory, social support and problem solving.

Weaknesses
- Although motivational interviewing will be used, it is not clear how it is integrated with the other theories.
- Unclear how contamination will be avoided.

5. Environment:
Strengths
- Excellent resources and facilities to support the study.

Weaknesses
- None noted

Protections for Human Subjects:
Acceptable Risks and/or Adequate Protections
- No concerns identified.

Data and Safety Monitoring Plan (Applicable for Clinical Trials Only):

Acceptable
  - No concerns were identified.

Inclusion of Women, Minorities and Children:
- Sex/Gender: Distribution justified scientifically
- Race/Ethnicity: Distribution justified scientifically
- Inclusion/Exclusion of Children under 21: Excluding ages < 21 justified scientifically
- No concerns identified.

Vertebrate Animals:
Not Applicable

Biohazards:
Not Applicable

Resubmission:
- The concerns raised in the previous review have been addressed.

Budget and Period of Support:

Recommend as Requested

- No concerns were identified.

CRITIQUE 3

Significance: 2
Investigator(s): 1
Innovation: 2
Approach: 2
Environment: 1

Overall Impact: This applications addresses diabetes in black and Latino adults; it uses mHealth with a clinical pharmacists and health coach. If proven effective it will provide a model for integrating pharmacists in chronic disease management and extend the use of telehealth in health disparities populations.

1. Significance:
Strengths
- It is a significant study given the morbidity and mortality associated with diabetes in racial and ethnic minority populations.

Weaknesses
- None noted

2. Investigator(s):
Strengths
- Dr. Gerber is an internist with expertise in conducting studies with diabetes population and in using mHealth and telehealth technology to impact health behaviors. He has developed mobile health applications that will be used on this study. He is supported by a team of health economists, pharmacists, behavioral health and social psychology researchers.

Weaknesses
- None noted.

3. Innovation:
Strengths
- Their prior study demonstrated that patients were reluctant to stay after a visit and discuss their care with a pharmacist because of time and they did not want to come back for a visit.
- However, videoconferencing with the pharmacist working remotely and a health coach in the home is an alternative and innovative solution which they are testing in this application.

Weaknesses

- None noted.

4. Approach:
Strengths

- A theoretical framework.
- There is a stronger rationale in the resubmission for testing video-conference rather than in-person visits.
- Demonstrated that it is acceptable to have health coaches in the home.
- Recruit from a wide range of practices.
- Build in one year of follow to assess maintenance.
- They have considered contingency plan for technical difficulties.

Weaknesses:

- Sustainability and implementation in other settings; is sustainability of the project beyond funding? The entire set up costs about $2000. The applications states a sustainability action plan will be developed. It would have been more informative if developed as part of the application not as a future endeavor.
- This works in a health care facility with a pharmacists on site, however, how would this work in community practices where pharmacists are lacking?

5. Environment:
Strengths

- University of Illinois Chicago team is well suited for this study with a wider range of support resources to enhance the implementation of this study

Weaknesses

- None noted

Protections for Human Subjects:

Acceptable Risks and/or Adequate Protections

- No concerns were identified.

Data and Safety Monitoring Plan (Applicable for Clinical Trials Only):

Acceptable

- No concerns were identified.

Inclusion of Women, Minorities and Children:

- Sex/Gender: Distribution justified scientifically
- Race/Ethnicity: Distribution justified scientifically
- Inclusion/Exclusion of Children under 21: Excluding ages < 21 justified scientifically
- No concerns identified.

Vertebrate Animals:
Not Applicable

Biohazards:
Not Applicable

Resubmission:

- The resubmission was responsive to the previous review.

Budget and Period of Support:

Recommend as Requested

- No concerns were identified.

THE FOLLOWING SECTIONS WERE PREPARED BY THE SCIENTIFIC REVIEW OFFICER TO SUMMARIZE THE OUTCOME OF DISCUSSIONS OF THE REVIEW COMMITTEE, OR REVIEWERS' WRITTEN CRITIQUES, ON THE FOLLOWING ISSUES:

PROTECTION OF HUMAN SUBJECTS (Resume): ACCEPTABLE

INCLUSION OF WOMEN PLAN (Resume): ACCEPTABLE

INCLUSION OF MINORITIES PLAN (Resume): ACCEPTABLE

INCLUSION OF CHILDREN PLAN (Resume): ACCEPTABLE

COMMITTEE BUDGET RECOMMENDATIONS: The budget was recommended as requested.

Footnotes for 1 R01 DK108141-01A1; PI Name: GERBER, BEN Steven

NIH has modified its policy regarding the receipt of resubmissions (amended applications). See Guide Notice NOT-OD-14-074 at http://grants.nih.gov/grants/guide/notice-files/NOT-OD-14-074.html. The impact/priority score is calculated after discussion of an application by averaging the overall scores (1-9) given by all voting reviewers on the committee and multiplying by 10. The criterion scores are submitted prior to the meeting by the individual reviewers assigned to an application, and are not discussed specifically at the review meeting or calculated into the overall impact score. Some applications also receive a percentile ranking. For details on the review process, see http://grants.nih.gov/grants/peer_review_process.htm#scoring.

MEETING ROSTER
Health Disparities and Equity Promotion Study Section
Healthcare Delivery and Methodologies Integrated Review Group
CENTER FOR SCIENTIFIC REVIEW

HDEP
02/04/2016 - 02/05/2016

CHAIRPERSON(S)

SOMMERS, MARILYN S, PHD, RN
LILLIAN S BRUNNER PROFESSOR OF MEDICAL SURGICAL NURSING
SCHOOL OF NURSING
UNIVERSITY OF PENNSYLVANIA
PHILADELPHIA, PA 19104

MEMBERS

ANTHONY, RENAISA SPRING, MD *
DEPUTY DIRECTOR
CENTER FOR REDUCING HEALTH DISPARITIES
COLLEGE OF PUBLIC HEALTH
UNIVERSITY OF NEBRASKA MEDICAL CENTER
OMAHA, NE 68198

ARCOLEO, KIMBERLY JOAN, PHD *
ASSOCIATE PROFESSOR
ASSOCIATE DEAN FOR RESEARCH
AND TRANSDISHIPLINARY SCHOLARSHIP
COLLEGE OF NURSING
OHIO STATE UNIVERSITY
COLUMBIA, OH 43210

BERRY, CAROLYN ANNE, PHD
ASSOCIATE PROFESSOR
DEPARTMENT OF POPULATION HEALTH
NEW YORK UNIVERSITY SCHOOL OF MEDICINE
NEW YORK, NY 10016

BOUTIN-FOSTER, CARLA, MD *
ASSOCIATE PROFESSOR
DIVISION OF GENERAL INTERNAL MEDICINE
DEPARTMENT OF MEDICINE
WEILL CORNELL MEDICAL COLLEGE
CORNELL UNIVERSITY
NEW YORK, NY 10021

CABASSA, LEOPOLDO J, PHD *
ASSOCIATE PROFESSOR
COLUMBIA UNIVERSITY
SCHOOL OF SOCIAL WORK
NEW YORK, NY 10032

CLARK, DANIEL O, PHD *
ASSOCIATE PROFESSOR
DEPARTMENT OF MEDICINE
INDIANA UNIVERSITY
INDIANAPOLIS, IN 46202

ELDER, JOHN P, PHD
PROFESSOR OF PUBLIC HEALTH
GRADUATE SCHOOL OF PUBLIC HEALTH
SAN DIEGO STATE UNIVERSITY
SAN DIEGO, CA 92123

FREUND, KAREN, MD *
PROFESSOR AND VICE CHAIR OF MEDICINE
TUFTS MEDICAL CENTER
TUFTS UNIVERSITY
BOSTON, MA 02111

GARCIA, VICTOR Q, PHD *
DISTINGUISHED UNIVERSITY PROFESSOR AND DIRECTOR
MID-ATLANTIC RESEARCH AND
TRAINING INSTITUTE (MARTI)
INDIANA UNIVERSITY OF PENNSYLVANIA
INDIANA, PA 15701

GRAVLEE, CLARENCE C *
ASSOCIATE PROFESSOR
DEPARTMENT OF ANTHROPOLOGY
UNIVERSITY OF FLORIDA
GAINESVILLE, FL 32611

HALL, LYNNE ANDERSON, BSN, DRPH, RN *
PROFESSOR
SCHOOL OF NURSING
UNIVERSITY OF LOUISVILLE
LOUISVILLE, KY 40207

HOWARD, VIRGINIA J, PHD *
PROFESSOR
DEPARTMENT OF EPIDEMIOLOGY
SCHOOL OF PUBLIC HEALTH
UNIVERSITY OF ALABAMA AT BIRMINGHAM
BIRMINGHAM, AL 35294

KAVANAUGH-LYNCH, MARION HELEN ELOISE, MD *
DIRECTOR
CALIFORNIA BREAST CANCER RESEARCH PROGRAM
UNIVERSITY OF CALIFORNIA
OAKLAND, CA 94612

KLASSEN, ANN CARROLL, PHD
PROFESSOR, ASSOCIATE DEAN FOR RESEARCH
DEPARTMENT OF COMMUNITY HEALTH AND PREVENTION
SCHOOL OF PUBLIC HEALTH
DREXEL UNIVERSITY
PHILADELPHIA, PA 19104

MARSHAL, MICHAEL P, PHD *
ASSOCIATE PROFESSOR
DEPARTMENT OF PSYCHIATRY
SCHOOL OF MEDICINE
UNIVERSITY OF PITTSBURGH
PITTSBURGH, PA 15213

MATTHEWS, ALICIA K, PHD
PROFESSOR
DEPARTMENT OF HEALTH SYSTEMS SCIENCES
COLLEGE OF NURSING
UNIVERSITY OF ILLINOIS AT CHICAGO
CHICAGO, IL 60612

MENON, USHA, FAAN, PHD, RN
ASSOCIATE DEAN FOR RESEARCH AND GLOBAL ADVANCES
COLLEGE OF NURSING
UNIVERSITY OF ARIZONA
TUCSON, AZ 85721

MUNOZ-LABOY, MIGUEL A, DPH *
ASSOCIATE PROFESSOR
SCHOOL OF SOCIAL WORK
TEMPLE UNIVERSITY
PHILADELPHIA, PA 19122

NUNEZ-SMITH, MARCELLA, MD *
ASSOCIATE PROFESSOR OF MEDICINE AND PUBLIC HEALTH
EQUITY RESEARCH AND INNOVATION CENTER
YALE UNIVERSITY
NEW HAVEN, CT 06520

ORTEGA, ALEXANDER N, PHD
PROFESSOR
UCLA CENTER FOR POPULATION HEALTH
AND HEALTH DISPARITIES
SCHOOL OF PUBLIC HEALTH
UNIVERSITY OF CALIFORNIA
LOS ANGELES, CA 90095

POSTON, WALKER S, PHD *
DEPUTY DIRECTOR AND SENIOR PRINCIPAL INVESTIGATOR
INSTITUTE FOR BIOBEHAVIORAL HEALTH RESEARCH
CENTER FOR FIRE, RESCUE AND EMS HEALTH RESEARCH
NATIONAL DEVELOPMENT
AND RESEARCH INSTITUTES NDRI
NEW YORK, NY 10010

PREWITT, THERESA ELAINE, DRPH *
ASSOCIATE PROFESSOR
DEPARTMENT OF HEALTH POLICY AND MANAGEMENT
DEP. DIR ARKANSAS CENTER FOR HEALTH DISPARATIES
FAY W. BOOZMAN COLLEGE OF PUBLIC HEALTH
UNIVERSITY OF ARKANSAS FOR MEDICAL SCIENCES
LITTLE ROCK, AR 72205

RADECKI BREITKOPF, CARMEN, PHD *
ASSOCIATE PROFESSOR
DIVISION OF HEALTH CARE POLICY AND RESEARCH
DEPARTMENT OF HEALTH SCIENCES RESEARCH
MAYO CLINIC COLLEGE OF MEDICINE
ROCHESTER, MN 55905

REGENSTEIN, MARSHA J, PHD
PROFESSOR
DEPARTMENT OF HEALTH POLICY
GEORGE WASHINGTON UNIVERSITY
WASHINGTON, DC 20052

SHUMWAY, MARTHA, PHD *
ASSOCIATE PROFESSOR
DEPARTMENT OF PSYCHIATRY
UNIVERSITY OF CALIFORNIA SAN FRANCISCO
SAN FRANCISCO, CA 94143

STROUP, ANTOINETTE MARIE, PHD *
ASSOCIATE PROFESSOR
DEPARTMENT OF EPIDEMIOLOGY
DIRECTOR, NEW JERSEY STATE CANCER REGISTRY
RUTGERS, THE STATE UNIVERSITY OF NEW JERSEY
SCHL OF PUBLIC HLTH & CANCER INST OF NEW JERSEY
NEW BRUNSWICK, NJ 08903

TEHRANIFAR, PARISA, DPH *
ASSISTANT PROFESSOR
EPIDEMIOLOGY
MAILMAN SCHOOL OF PUBLIC HEALTH
COLUMBIA UNIVERSITY
NEW YORK, NY 10032

THOMAS, STEPHEN B, PHD
DIRECTOR, MARYLAND CENTER FOR HEALTH EQUITY
PROFESSOR, DEPARTMENT OF HEALTH SERVICES ADMINISTRATION, SCHOOL OF PUBLIC HEALTH
UNIVERSITY OF MARYLAND, COLLEGE PARK
COLLEGE PARK, MD 20742

VIDRINE, JENNIFER IRVIN, PHD *
PEGGY AND CHARLES STEPHENSON ENDOWED CHAIR IN CANCER DEPUTY DIRECTOR FOR TOBACCO RESEARCH AND DIRECTOR, OKLAHOMA TOBACCO RESEARCH CENTER
STEPHENSON CANCER CENTER
ASSOCIATE PROFESSOR, DEPARTMENT OF FAMILY AND PREVENTIVE MEDICINE
UNIVERSITY OF OKLAHOMA HEALTH SCIENCES CENTER
OKLAHOMA CITY, OK 73104

WHITT-GLOVER, MELICIA C, PHD
PRESIDENT AND CHIEF EXECUTIVE OFFICER
GRAMERCY RESEARCH GROUP, LLC
WINSTON-SALEM, NC 27106

WILLIAMS, KRISTINE N, BSN, PHD *
SALLY MATHIS HARTWIG PROFESSOR
GERONTOLOGICAL NURSING
COLLEGE OF NURSING
THE UNIVERSITY OF IOWA
IOWA CITY, IA 52242

WONG, FRANK Y, PHD
ASSOCIATE PROFESSOR
DEPARTMENT OF BEHAVIORAL SCIENCES
AND HEALTH EDUCATION
ROLLINS SCHOOL OF PUBLIC HEALTH
EMORY UNIVERSITY
ATLANTA, GA 30322

WU, BEI, PHD *
PAULINE GRATZ PROFESSOR OF NURSING AND
PROFESSOR OF GLOBAL HEALTH
DIRECTOR FOR INTERNATIONAL RESEARCH
SCHOOL OF NURSING
DUKE UNIVERSITY
DURHAM, NC 27710

MAIL REVIEWER(S)

CHRISTENSEN, KATHEE M, PHD
PROFESSOR
DEPARTMENT OF COMMUNICATIVE DISORDERS
SAN DIEGO UNIVERSITY
SAN DIEGO, CA 92182

KARNIK, NIRANJAN, MD, PHD
ASSOCIATE PROFESSOR
DEPARTMENT PSYCHIATRY
RUSH UNIVERSITY MEDICAL CENTER
CHICAGO, IL 60612

SMITH, TRISTRAM H, PHD
PROFESSOR
DEPARTMENT OF PEDIATRICS
SCHOOL OF MEDICINE AND DENTISTRY
UNIVERSITY OF ROCHESTER
ROCHESTER, NY 14618

SCIENTIFIC REVIEW OFFICER

BELLINGER, JESSICA, PHD
CENTER FOR SCIENTIFIC OF REVIEW
NATIONAL INSTITUTES OF HEALTH
BETHESDA, MD 20892

OLUFOKUNBI SAM, DELIA, PHD
SCIENTIFIC REVIEW OFFICER
CENTER FOR SCIENTIFIC REVIEW
NATIONAL INSTITUTES OF HEALTH
BETHESDA, MD 20892

EXTRAMURAL SUPPORT ASSISTANT

BARTLETT, VALERIE
EXTRAMURAL SUPPORT ASSISTANT
CENTER FOR SCIENTIFIC REVIEW
NATIONAL INSTITUTES OF HEALTH
BETHESDA, MD 20892

* Temporary Member. For grant applications, temporary members may participate in the entire meeting or may review only selected applications as needed.

Consultants are required to absent themselves from the room during the review of any application if their presence would constitute or appear to constitute a conflict of interest.
